# The Third Spectrum of Gold (Au III)

**DOI:** 10.6028/jres.064A.046

**Published:** 1960-12-01

**Authors:** Laura Iglesias

## Abstract

The spark spectrum of gold has been photographed in a helium atmosphere from 500 A to 6600 A. About 500 lines have been assigned to the third spectrum, Au III, and separated from those belonging to different stages of ionization, by observation of the polarity of the lines. Sixty two levels have been found: 17 even levels, arising from the 5*d*^9^ and *5d*^8^ 6*s* configurations; and 45 odd levels, belonging to the *5d*^8^ *6p* and 5*d*^7^ 6s 6*p* configurations. All of the expected levels from the configurations *5d*^9^, 5*d*^8^ 6s and 5*d*^8^ 6*p* have been identified except for the very high terms based on the *5d*^8^(^1^S) core of Au IV. With these levels it was possible to classify 256 lines.

## 1. Introduction

The first and second spectra of gold were studied some years ago, but apparently no attempt has been made to study the structure of the third spectrum, (An III).

As in most spectra of the third long period, the Au III spectrum reveals a coupling intermediate between *LS*-coupling and *jj*-coupling since the spin-spin interaction and the spin-orbit interaction are both large. It approaches *jj*-coupling rather than *LS*, especially in the case of levels belonging to the *5d*^8^ 6*p* configuration.

It is, therefore, very difficult to describe the observed levels rigorously in any one scheme. Accordingly, the even levels and the odd quartet levels based on 5*d*^8^(^3^F), which are perhaps closer to *LS*-coupling, have been given names in that system, and the other odd levels have been given numbers simply for identification.

## 2. Analysis and Results

The analysis is based mainly on plates taken on the 2-m vacuum spectrograph at Princeton, in the wavelength region 500 to 2200 A.

The light source was a condensed spark in helium at a pressure of approximately 1 atm.

In order to excite the third spectrum and to be able to differentiate it from the other stages of ionization, the same technique was used that A.G. Shenstone has described in his paper on Ni III.^2^ Initially, the exposures were limited by the clogging of the slit by gold particles, sputtered from the spark source. Apparently gold has a pronounced tendency to sputter and the complete clogging of the slit took only one or two min, instead of the half hour or more which is commonly found with other metals. To avoid this limitation, it was necessary to fill the body of the spectrograph with helium to atmospheric pressure, so that there would be no flow through the slit. For the spectrograms taken above 1200 A, this was not necessary since the lithium fluoride window separated the spectrograph from the source.

The spectrograms were photographed on Ilford Q–2 plates and the exposures varied from 1 to 15 min. The impurity lines of nitrogen, oxygen, and carbon present on the plates were used as standards. The estimated probable error of the experimental wavelengths entered in table 1, column 1, is ±0.01 A.

To complete the observations, other plates were taken on the 21-ft grating in a Wadsworth mounting by using Eastman 103aO plates, 103aD, and 103aF according to the region. About 500 lines in all were assigned to the third spectrum.

The lowest levels should be the ^2^D_2½_ and D_1½_ of the *5d*^9^ cofinguration, and by comparison with the Au I and Au II spectra,^3^ the interval between them should be about −13000 cm^−1^. The equivalent interval in Au I and II is as follows:

Au I5d96s22D212−2D112—12274Au II5d9( 2D)6s3D3−3D1—12725

In fact, we found it to be −12694.0 in Au III.

In order to have some idea about the position of the levels of the *5d*^8^ 6*s* and *5d*^8^ 6*p* configurations we plotted Ir I and Pt II and the analogous spectra of the second long period Rh I, Pd II and Ag III, using the values given in “Atomic Energy Levels”.^3^ To find the levels the usual method of searching for equal wave-number differences was used.

**Figure 1:**
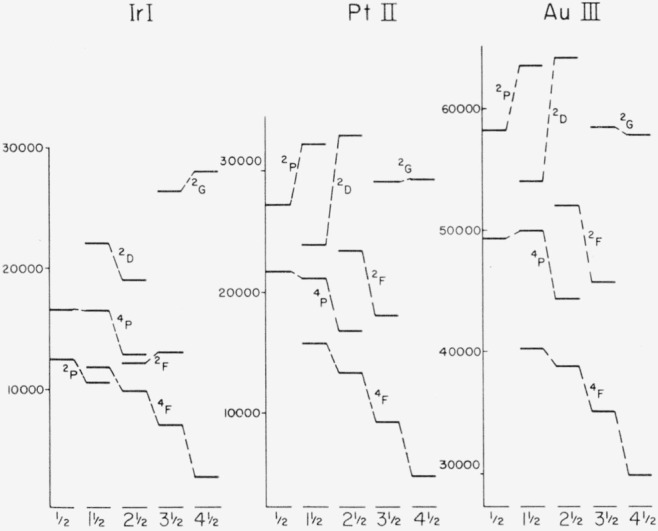
Configuration 5d^8^ 6s in *Ir I, Pt II*, and *Au III*.

A remarkable similarity was found in the case of the terms arising from the *5d*^8^ 6*s* configuration. This relationship is shown in figure 1 where the relative values of the levels of Ir I, Pt II and Au III are plotted against *J*-values.

Based on this similarity and the reason explained above, a tentative *LS*-term assignment has been made and is given in table 2, where all the known even levels are arranged in increasing numerical order.

In the case of the odd configuration *5d*^8^ 6*p*, the levels are so mixed in character that it is meaningless to group them into terms. A very tentative designation based on combinations and intensities appears in table 3, but these names, except for the quartet levels based on 5*d*^8^(^3^F), are not used in the line list of table 1. Instead, the levels are assigned numbers for identification.

In table 3, 43 levels of the *5d*^8^ 6*p* configuration are listed, plus two more belonging to the *5d*^7^ 6*s* 6*p* configuration.

The configurations 5*d*^9^, 5*d*^8^ 6*s* and *5d*^8^ 6*p* are complete except for the usual failure to find the levels based on *5d*^8^(^1^S) of Au IV.

With the 62 levels found, it was possible to classify 256 of the 500 observed lines attributable to Au III as they appear in table 1.

## Figures and Tables

**Table 1:** Identified lines of *Au III*

λ(vac.)	Intensity and character	Wave No.	Combination

*A*		*cm*^−1^	
751.554	2	133057.6	a2D212—42212°
763.497	20	130976.3	a2D212—40312°
779.728	30	128249.8	a2D212—38112°
788.783	30	126777.6	a4F412—44312°
811.394	25	123244.7	a4F312—45212°
811.831	40	123178.3	a2D212—32112°
816.129	3	122529.6	a2D212—31212°
817.96	50	122256	a2D112—43212°
820.053	40	121943.3	a2D212—29112°
820.846	5	121825.5	a2D212—28312°
823.338	10	121456.8	a4F312—44312°
833.149	80	120026.5	a2D212—27212°
836.804	15	119502.3	a4F212—45212°
843.454	100	118560.1	a2D212—26112°
845.138	100	118323.9	a2D212—25312°
847.619	5	117977.5	a4F112—45212°
849.546	5	117709.9	a4F212—44312°
855.495	80	116891.4	a2D212—24112°
859.891	80	116293.8	a2D212—23312°
863.425	80	115817.8	a2D112—39212°
883.782	50	113150.1	a2D112—35012°
885.906	60	112878.8	a2D212—z4F212°
901.025	80	110984.6	a2D212—16312°
905.105	30	110484.4	a2D112—32112°
910.446	80	109836.3	a2D112—31212°
911.470	20	109712.9	a2D112—30012°
914.175	5*h*	109388.2	a2D212—z4F112°
915.338	8	109249.3	a2D112—29112°
924.037	80	108220.8	a2D212—14212°
931.675	10	107333.6	a2D112—27212°
941.050	20	106264.3	a2D212—12112°
944.581	50	105867.0	a2D112—26112°
945.099	200	105809.0	a2D212—z4F312°
959.720	15	104197.0	a2D112—24112°
973.893	10	102680.7	a2D112—21212°
977.318	40	102320.8	a2D212—z4G312°
989.408	25	101070.5	a2D112—19012°
998.156	20	100184.7	a2D112—z4F212°
1034.206	60	96692.5	a2D112—z4F112°
1040.650	100	96093.8	a2D212—z4G212°
1044.497	80	95739.9	a2D212—z4D112°
1046.825	80	95526.9	a2D112—14212°
1054.192	40	94859.4	a2D112—13212°
1086.110	10	92072.5	a4F412—28312°
1123.172	5	89033.5	a2D112—z4D212°
1199.022	25	83401.3	a2D112—z4G212°
1204.155	30	83045.8	a2D112—z4D112°
1229.364	30	81342.9	a4P212—34112°
1231.060	20	81230.8	a2F412—16312°
1231.266	30	81217.2	a2F312—23312°
1239.961	100*w*	80647.7	a4F312—22412°
1254.996	30	79681.5	a4F112—27212°
1277.442	5	78281.4	a4P112—38112°
1278.514	100	78215.8	a4F112—26112°
1280.903	50	78069.9	a4F212—24112°
1285.302	50	77802.7	a4F312—z4F212°
1290.029	30	77517.6	a4P212—29112°
1290.358	40	77497.9	a4P112—37112°
1290.795	50	77471.6	a4F212—23312°
1291.979	60	77400.6	a4P212—28312°
1306.317	5	76551.1	a4F212—21212°
1306.409	5	76545.7	a4F112—24112°
1307.988	20	76453.3	a2F212—39212°
1308.776	10	76407.3	a4P012—35012°
1309.440	40	76368.5	a2G312—43212°
1314.825	100	76055.7	a4F412—z4F312°
1322.728	30	75601.3	a4P212—27212°
1326.105	12	75408.8	a2F212—37112°
1336.700	200	74811.1	a4F412—z4F412°
1341.660	180	74534.5	a2G412—41412°
1346.129	12	74287.1	a2F312—27212°
1348.873	100	74136.0	a4P212—26112°
1349.200	30	74118.0	b2D112—38112°
1350.302	150	74057.5	a4F212—z4F212°
1353.200	50	73898.9	a4P212—25312°
1355.598	150	73768.2	a2G312—41412°
1356.109	150	73740.4	a4P012—32112°
1362.038	80	73419.4	a4F112—19012°
1365.372	500	73240.1	a4F412—z4G512°
1365.949	10	73209.2	a4P112—32112°
1366.874	20	73159.6	a2G412—40312°
1367.149	200	73144.9	a4F312—14212°
1377.708	180	72584.3	a2F312—25312°
1378.048	10	72566.4	a4F412—z4G312°
1378.166	20	72560.2	a4P112—31212°
1378.655	150	72534.5	a4F112—z4F212°
1379.222	50	72504.6	a4P012—29112°
1379.951	150	72466.3	a4P212—24112°
1380.498	125	72437.6	a4P112—30012°
1381.338	200	72393.6	a2G312—40312°
1385.763	300	72162.4	a4F212—16312°
1389.388	100	71974.1	a4P112—29112°
1391.441	180	71867.9	a4P212—23312°
1395.971	180	71634.7	b2D112—34112°
1402.878	100	71282.0	a2P112—43212°
1406.079	20	71119.7	a2F212—32112°
1409.472	225	70948.5	a4P212—21212°
1413.779	250	70732.4	a4F312—z4F312°
1414.247	100	70709.0	b2D212—43212°
1417.111	100	70566.1	a4F212—z4F112°
1417.368	125	70553.3	a2F312—23312°
1419.023	60	70471.0	a2F212—31212°
1427.393	150	70057.8	a4P112—27212°
1428.907	300	69983.6	a2F312—22412°
1430.037	250	69928.3	a2G312—39212°
1433.344	275	69766.9	a2F212—28312°
1435.784	250	69648.3	a2G412—36512°?
1436.088	80	69633.6	a2F312—21212°
1436.802	30	69599.0	a2F312—20312°
1439.100	300	69487.9	a4F312—z4F412°
1441.173	200	69387.9	a2P112—42212°
1446.334	150	69140.3	a2P012—37112°
1446.701	80	69122.8	a4P012—26112°
1448.393	250	69042.0	a4F112—z4F112°
1453.173	50	68814.9	b2D212—42212°
1454.927	250	68732.0	a4F212—13212°
1462.048	40	68397.2	b2D112—31212°
1464.692	100	68273.7	b4D112—30012°
1471.281	150	67968.0	a2F212—27212°
1473.279	80	67875.8	a2F112—14212°
1474.707	100	67810.0	a2D112—29112°
1481.066	150	67518.9	a2P012—35012°
1482.510	30	67453.2	a4P012—24112°
1482.775	30	67441.1	a4F212—12112°
1487.133	300	67243.5	a4F312—z4G312°
1487.906	250	67208.5	a4F112—13212°
1489.446	200	67139.0	a2F312—z4F212°
1492.829	( )	66986.9	a4F212—z4F312°
1494.266	20	66922.5	a4P112—24112°
1500.334	250	66651.8	a4F312—z4D212°
1502.441	200	66558.3	a4P212—16312°
1503.716	200	66501.9	a2F212—26112°
1517.049	10	65917.4	a4F112—12112°
1528.941	40	65404.7	a4P112—21212°
1540.258	80	64924.2	a2G312—33412°?
1541.978	100	64851.8	a2P012—32112°
1542.212	80	64841.9	a2P112—39212°
1548.473	100	64579.7	a2P112—38112°
1554.580	80	64326.0	a4P012—19012°
1556.793	20	64234.6	a2F212—23312°
1560.550	30	64080.0	a2P012—30012°
1562.328	80	64007.0	{a2G412—28312°b2D212—38112°
1562.429	80	64002.9	a4F112—9012°
1563.826	20	63945.7	a2G312—31212°
1567.512	200	63795.4	{a4P112—19012°b4P212—14212°
1571.901	80	63617.2	a2P012—29112°
1574.855	200	63497.9	a2F212—z4G312°
1579.413	200	63314.7	a2F212—21212°
1580.277	20	63280.0	a2F212—20312°
1581.226	20	63242.0	a2G312—28312°
1584.074	150	63128.4	a2P212—13212°
1589.559	200	62910.5	a4P112—z4F212°
1589.680	80	62905.7	a4F212—z4D212°
1593.394	150	62759.1	b2D112—24112°
1600.496	200	62480.6	a2F312—14212°
1608.348	40	62175.6	a2P112—35012°
1610.390	60	62096.7	a2P112—34112°
1617.137	250	61837.7	a4P212—12112°
1617.761	100	61813.8	a2F312—13212°
1621.913	500	61655.6	a2F412—z4G412°
1625.384	10	61523.9	b2D212—34112°
1629.116	300*d*	61383.0	{a4P212—z4F312°a4F112—z4D212°
1632.891	60	61241.1	b2D112—21212°
1638.876	250	61017.6	a2F312—z4G212°
1644.189	100	60820.3	a2F212—z4F212°
1652.733	250	60505.8	b2G412—25312°
1664.778	250	60068.1	a2F312—z4F312°
1668.098	100	59948.5	a4P012—z4F112°
1673.919	125	59740.0	a2G312—25312°
1676.957	40	59631.8	b2D112—19012°
1693.917	1000	59034.8	a4F412—z4D312°
1697.081	150	58924.7	a2F212—16312°
1698.970	200	58859.2	a2P112—31212°
1699.990	200	58823.9	a2F312—z4F412°
1702.235	200	58746.3	b2D112—z4F212°
1707.508	100	58564.9	a2P012—24112°
1710.125	250	58475.2	a2G412—23312°
1715.670	200	58286.3	b2D212—31212°
1716.697	100	58251.4	a4P112—14212°
1717.820	300	58213.3	a4F112—z4D012°
1726.952	5	57905.5	a2G412—22412°
1727.281	500	57894.5	a4P212—z4G312°
1733.140	100*d*	57698.9	b2D212—29112°
1736.590	60	57584.1	a4P112—13212°
1738.484	300	57521.4	a2G412—20312°
1744.346	150	57328.1	a2F212—z4F112°
1745.098	40	57303.4	a4P212—z4D212°
1746.037	500	57272.5	a4F212—z4G212°
1750.095	30	57139.9	a2G312—22412°
1756.917	500	56917.9	a4F212—z4D112°
1759.800	20	56824.6	a4P012—12112°
1760.881	60	56789.7	a2G312—21212°
1761.947	500	56755.4	a2G312—20312°
1767.415	300	56579.8	a2F312—z4G312°
1774.419	100	56356.5	a2P112—27212°
1775.166	800	56332.8	a4F312—z4G412°
1776.396	200	56293.8	a4P112—12112°
1780.571	100	56161.8	a2F212—14212°
1786.106	300	55987.7	a2F312—z4D212°
1792.653	150	55783.2	b2D212—27212°
1793.762	500	55748.8	a4F112—z4G212°
1801.982	200	55494.4	a2F212—13212°
1805.235	400	55394.4	a4F112—z4D112°
1809.811	100	55254.4	b2D112—z4F112°
1821.169	400	54909.8	a4P012—9012°
1821.801	20	54890.7	a2P112—26112°
1841.019	20	54317.7	b2D212—26112°
1844.889	400	54203.8	a2F212—12112°
1848.833	150	54088.2	b2D112—14212°
1849.088	50	54080.7	b2D212—25312°
1860.484	40	53749.5	a2F212—z4F312°
1861.799	500	53711.5	a4F312—z4D312°
1871.922	150	53421.0	b2D112—13212°
1880.911	30	53165.7	a2G412—16312°
1899.405	60	52648.1	b2D212—24112°
1918.278	150	52130.1	b2D112—12112°
1932.038	100	51758.8	b4P112—z4D212°
1934.114	60	51703.3	a2P112—21212°
1935.416	100	51668.2	a4P212—z4G212°
1948.792	200	51313.8	a4P212—z4D112°
1958.472	100	51060.2	a2P012—z4F112°
1985.951	20*d*	50353.7	a2F012—z4G212°
1989.631	400	50260.6	a2F212—z4G312°
1996.853	150	50078.8	a2P112—18012°
2041.435	60	48969.4	a2G312—13212°
2055.459	60	48635.4	b2D212—z4F212°
2083.092	300	47990.3	a4G412—z4F312°
2085.452	80	47936.0	a2P012—12112°
2100.392	20	47595.0	b1D112—z4D212°
2116.879	2	47224.4	a2G312—z4F312°
2159.085	100	46301.2	a4P012—z4D112°
2167.332	80	46125.2	a4P112—z4G212°
2172.200	200	46021.8	a2P012—9012°
2184.108	100	45770.9	a4P112—z4D112°
2186.673	50	45717.3	a2P112—z4F112°
2188.966	500	45669.4	a2F312—z4G412°
2253.448	40	44362.7	a4P212—z4D312°
2270.217	10	44035.2	a2F212—z4G212°
2278.045	5	43883.7	a2P112—13212°
2288.626	40	43680.8	a2F212—z4D112°
2308.200	10	43310.5	b2D212—13212°
2322.267	300	43048.1	a2F312—z4D312°
2347.105	20	42592.6	a2P112—12112°
2379.106	10	42019.8	b2D212—12112°
2382.403	100	41961.6	b2D112—z4G212°
2402.706	150	41607.1	b2D112—z4D112°
2405.118	150	41565.3	b2D212—z4F312°
2625.522	10*d*	38076.3	b2D212—z4G312°
2665.159	10	37510.1	a2G312—z4G212°
2666.994	10	37484.3	b2D212—z4D212°
2721.835	50	36729.0	a2F212—z4D312°
3117.339	10	32069.3	a2P112—z4D112°
3138.730	10	31850.8	b2D212—z4G212°
3174.057	20	31496.3	b2D212—z4D112°
3227.991	100	30970.0	a2G412—z4G312°
3309.856	100	30204.1	a2G312—z4D312°

NOTE.—*h* = hazy; *w*=wide; ( ) = masked by another line; *d*=doublo.

**Table 2:** Even levels of *Au III*

Electron structure	Possible designation	*J*	Level	Interval

*5d*^9^	*a* ^2^D	2½	0.0	−12694.0
5*d^9^*	*a* ^2^D	1½	12694.0	
5*d*^8^(^3^F_4_)6*s*	*a* ^4^F	4½	29753.6	−5323.1 −3745.5 −1523.4
5*d*^8^(^3^F_4_)6*s*	*a* ^4^F	3½	35076.7	
5*d*^8^(^3^F_3_)6*s*	*a* ^4^F	2½	38822.2	
5*d*^8^(^3^F_2_)6*s*	*a* ^4^F	1½	40345.6	
5*d*^8^(^3^P_2_)6*s*	*a* ^4^P	2½	44425.9	
5*d*^8^(^3^F_3_)6*s*	*a* ^2^F	3½	45740.5	
5*d*^8^(^3^P_1_)6*s*	*a* ^4^P	0½	49438.9	
5*d*^8^(^3^P_2_)6*s*	*a* ^4^P	1½	49969.4	
5*d*^8^(^3^F_2_)6*s*	*a* ^2^F	2½	52059.6	
5*d*^8^(^1^D_2_)6*s*	*b* ^2^D	1½	54133.2	
5*d*^8^(^1^G_4_)6*s*	*a* ^2^G	4½	57818.6	
5*d*^8^(^3^P_0_)6*s*	*a* ^2^P	0½	58327.1	
5*d*^8^(^1^G_4_)6*s*	*a* ^2^G	3½	58584.6	
5*d*^8^(^3^P_1_)6*s*	*a* ^2^P	1½	63670.9	
5*d*^8^(^1^D_2_)6*s*	*b* ^2^D	2½	64244.0	

**Table 3:** Odd levels of *Au III*

Electron structure	*Possible designation*	*J*	Level	*Interval*

5*d*^8^(^3^F)6*p*	*z* ^4^D°	1°	3½	*88788*.*5*
5*d*^8^(^3^F)6*p*	*z* ^4^G°	2°	4½	*91409.4*
5*d*^8^(^3^F)6*p*	*z* ^4^D°	3°	1½	*95740.0*
5*d*^8^(^8^F)6*p*	*z* ^4^G°	4°	2½	*96094*.*5*
5*d*^8^(^3^F)6*p*	*z* ^4^D°	5°	0½	*98559.1*
5*d*^8^(^3^F)6*p*	*z* ^4^D°	6°	2½	*101728.2*
5*d*^8^(^3^F)6*p*	*z* ^4^G°	7°	3½	*102320.2*
5*d*^8^(^3^F)6*p*	*z* ^4^G°	8°	5½	*102993.7*
5*d*^8^(^3^P)6*p*	^4^P°	9°	0½	*104348.3*
5*d*^8^(^3^F)6*p*	*z* ^4^F°	10°	4½	*104564.6*
5*d*^8^(^3^F)6*p*	*z* ^4^F°	11°	3½	*105809.1*
5*d*^8^(^3^P)6*p*	^4^P°	12°	1½	*106263.1*
5*d*^8^(^3^P)6*p*	^4^P°	13°	2½	*107554.2*
5*d*^8^(^3^F)6*p*	^2^D°	14°	2½	*108221.2*
5*d*^8^(^3^F)6*p*	*z* ^4^F°	15°	1½	*109387.6*
5*d*^8^(^3^P)6*p*	^4^D°	16°	3½	*110984.1*
3*d*^8^(^3^F)6*p*	*z* ^4^F°	17°	2½	*112879.6*
5*d*^8^(^3^P)6*p*	^2^S°	18°	0½	*113749.9*
5*d*^8^(^1^D)6*p*	^2^P°	19°	0½	*113764.9*
5*d*^8^(^1^G)6*p*	^2^F°	20°	3½	*115339.9*
5*d*^8^(^3^F)6*p*	^2^F°	21°	2½	*115374.2*
5*d*^8^(^3^F)6*p*	^2^G°	22°	4½	*115724.2*
5*d*^8^(^3^F)6*p*	^2^F°	23°	3½	*116293.8*
5*d*^8^(^3^P)6*p*	^4^S°	24°	1½	*116892.1*
5*d*^8^(^1^D)6*p*	^2^F°	25°	3½	*118324.6*
5*d*^8^(^3^F)6*p*	^2^D°	26°	1½	*118561.7*
5*d*^8^(^3^P)6*p*	^4^D°	27°	2½	*120027.3*
5*d*^8^(^3^F)6*p*	^2^G°	28°	3½	*121826.4*
5*d*^8^(^1^D)6*p*	^2^P°	29°	1½	*121943.5*
5*d*^8^(^3^P)6*p*	^4^D°	30°	0½	*122407.0*
5*d*^8^(^1^D)6*p*	^2^F°	31°	2½	*122530.3*
5*d*^8^(^3^P)6*p*	^4^D°	32°	1½	*123179.0*
5*d*^8^(^1^G)6*p*	^2^H°	33°	4½	*123508.8*?
5*d*^8^(^1^D)6*p*	^2^D°	34°	1½	*125767.9*
5*d*^8^(^3^P)6*p*	^2^P°	35°	0½	*125846.2*
5*d*^8^(^1^G)6*p*	^2^H°	36°	5½	*127467.0*?
5*d*^8^(^3^P)6*p*	^2^D°	37°	1½	*127467.6*
5*d*^8^(^3^P)6*p*	^2^P°	38°	1½	*128250.9*
5*d*^8^(^1^G)6*p*	^2^F°	39°	2½	*128512.7*
5*d*^8^(^1^G)6*p*	^2^G°	40°	3½	*130978.2*
5*d*^8^(^1^G)6*p*	^2^G°	41°	4½	*132353.0*
5*d*^8^(^3^P)6*p*	^2^D°	42°	2½	*133058.9*
5*d*^8^(^1^D)6*p*	^2^D°	43°	2½	*134953.0*
5*d^7^ 6s* 6*p*		44°	3½	*156532.0*
5*d*^7^ 6*s* 6*p*		45°	2½	*158323.1*

## Footnotes

2 A.G. Shenstone, J. Opt. Soc. Am. **44**, 749 (1954).

3 C.E. Moore, Atomic Energy Levels, NBS Circ. 467 (1958).

